# Experimental evaluation of ecological principles to understand and modulate the outcome of bacterial strain competition in gut microbiomes

**DOI:** 10.1038/s41396-022-01208-9

**Published:** 2022-02-24

**Authors:** Rafael R. Segura Munoz, Sara Mantz, Ines Martínez, Fuyong Li, Robert J. Schmaltz, Nicholas A. Pudlo, Karthik Urs, Eric C. Martens, Jens Walter, Amanda E. Ramer-Tait

**Affiliations:** 1 Department of Food Science and Technology, University of Nebraska-Lincoln, Lincoln, Nebraska USA; 2 Nebraska Food for Health Center, University of Nebraska-Lincoln, Lincoln, Nebraska USA; 3 Department of Agricultural, Food and Nutritional Science, University of Alberta, Edmonton, Canada; 4 Department of Biological Sciences, University of Alberta, Edmonton, Canada; 5 Department of Infectious Diseases and Public Health, Jockey Club College of Veterinary Medicine and Life Sciences, City University of Hong Kong, Kowloon, Hong Kong SAR China; 6 Department of Microbiology and Immunology, University of Michigan Medical School, Ann Arbor, Michigan USA; 7 APC Microbiome Ireland, School of Microbiology, and Department of Medicine, University College Cork, Cork, Ireland

**Keywords:** Microbial ecology, Microbiome

## Abstract

It is unclear if coexistence theory can be applied to gut microbiomes to understand their characteristics and modulate their composition. Through experiments in gnotobiotic mice with complex microbiomes, we demonstrated that strains of *Akkermansia muciniphila* and *Bacteroides vulgatus* could only be established if microbiomes were devoid of these species. Strains of *A. muciniphila* showed strict competitive exclusion, while *B. vulgatus* strains coexisted but populations were still influenced by competitive interactions. These differences in competitive behavior were reflective of genomic variation within the two species, indicating considerable niche overlap for *A. muciniphila* strains and a broader niche space for *B. vulgatus* strains. Priority effects were detected for both species as strains’ competitive fitness increased when colonizing first, which resulted in stable persistence of the *A. muciniphila* strain colonizing first and competitive exclusion of the strain arriving second. Based on these observations, we devised a subtractive strategy for *A. muciniphila* using antibiotics and showed that a strain from an assembled community can be stably replaced by another strain. By demonstrating that competitive outcomes in gut ecosystems depend on niche differences and are historically contingent, our study provides novel information to explain the ecological characteristics of gut microbiomes and a basis for their modulation.

## Introduction

The gut microbiota is considered an important aspect of host health, influencing digestion, immune system development, and pathogen invasion [1–7]. Moreover, numerous studies have documented differences in microbiome composition and function between healthy and diseased humans and animals [8, 9]. Strategies aimed at modulating and restoring the ecological and physiological features of the gut microbiome have therefore gained much momentum [10, 11]. Considering the complexity of the gut ecosystem, successful modulation of gut microbiomes is likely to require the application of ecological theory [12–14].

The introduction of live microbes, either as single strains (e.g., probiotics, live biotherapeutics) or complex mixtures (e.g., fecal microbiota transplants), into the gut ecosystem represents one approach to modifying the microbiome. However, the ecological requirements for sustained long-term colonization (i.e., engraftment) of orally administered live microbial products are poorly understood [15]. Recent evidence suggests that engraftment may depend on the pre-treatment microbiome composition, especially the absence of closely related species [16–19]. We [17, 18] and others [16, 20–22] have shown that the resident microbiome influences engraftment of incoming species, likely through competitive exclusion where newly-arriving species cannot coexist with established species if they occupy exactly the same niche (and are competing for identical resources) [23]. In particular, Maldonado and colleagues showed that persistence of the probiotic *Bifidobacterium longum* AH1206 in select study participants was associated with low abundance of *B. longum* species [17]. Lack of colonization in other participants may thus be explained by competitive exclusion [23]. However, these findings are based on associations, and it has not been established if competitive exclusion is in fact the causative factor that determines engraftment. Indeed, not all outcomes from microbiome-based interventions are consistent with competitive exclusion and instead indicate coexistence of related strains in fecal transplants [20, 24]. These discrepancies illustrate that the ecological factors governing engraftment are complex, insufficiently understood, and likely extend beyond the competitive exclusion principle [13].

Modern coexistence theory suggests that competitive exclusion and coexistence of species or strains are determined by equalizing mechanisms that reduce fitness differences among members and stabilizing mechanisms that decrease competition through niche differences (i.e., resource partitioning) [25, 26]. Competitive interactions are further historically contingent, meaning that order of arrival of a species into an ecosystem can result in priority effects that alter the outcome of species interactions (e.g., by benefiting early colonizers over late colonizers) [26]. For gut ecosystems, competitive exclusion has been demonstrated for isogenic bacterial strains orally administered to germ-free mice [27, 28]. However, such an experiment represents an extreme condition where the competing strains are essentially identical and have maximum niche overlap. Strains in natural communities possess genetic and trait variation, and strains used as live microbial products are likely to differ from resident microbiota members. To what degree contemporary niche and coexistence theory applies to interactions among members of the gut microbiota and the introduction of microbes into gut microbiomes has not been experimentally tested.

Here, we performed systematic experiments in gnotobiotic mice to test the applicability of coexistence theory to the stable establishment of gut microbes and to determine if such information can be utilized to stably modulate the gut microbiota at the strain level. We selected two species of gut bacteria, *Akkermansia muciniphila* and *Bacteroides vulgatus*, based on their importance in modulating host metabolism [29, 30] and immunity [31–33]. To study niche occupancy and intraspecific competition under close-to-natural but strictly controlled conditions, we utilized gnotobiotic mice colonized with complex microbiomes with and without *A. muciniphila* and *B. vulgatus*. We specifically tested (i) to what degree the absence of a species determines colonization, (ii) if colonization can be prevented or altered by prior introduction of another strain of a species, and (iii) the effect of colonization order on coexistence between strains of the same species. Finally, we applied the information gained from these experiments to design a subtractive antibiotic strategy with the aim of removing an *A. muciniphila* strain from an assembled community and replacing it with a different strain.

## Materials and methods

### Bacteria

Bacterial strains used in this study were: *Akkermansia muciniphila* BAA-835 (American Tissue Culture Collection, Manassas, VA) isolated from human feces, *A. muciniphila* YL44 (Leibniz Institute DSMZ, Braunschweig, Germany) isolated from C57BL/6 J mouse feces, *Bacteroides vulgatus* ATCC 8482 (American Tissue Culture Collection), and *B. vulgatus* RJ2H1 [18]. *A. muciniphila* strains were cultured in 10 mL Brain Heart Infusion (BHI) medium (BD, Sparks, MD) supplemented with 0.3% (w/v) mucin from porcine stomach Type II (Sigma-Aldrich, St. Louis, MO) and 0.5% yeast extract (BD, Sparks, MD); this medium is referred to as BHIm in this manuscript. *B. vulgatus* strains were grown in 10 mL BHI medium supplemented with menadione (0.001 g/L, Sigma-Aldrich), hemin (0.005 g/L, Sigma-Aldrich), and an additional 0.5% yeast extract (BD, Sparks, MD); this medium is referred to as BHIs throughout this manuscript. Carbohydrate growth arrays for *B. vulgatus* 8482 and RJ2H1 were performed as previously described [34, 35].

To inoculate mice, one vial of each bacterial strain was retrieved from −80 °C storage and the entire contents were struck onto a plate containing BHIm with agar (1% w/v). Plates were incubated at 37 °C under anaerobic conditions for either three days for *B. vulgatus* strains or five days for *A. muciniphila* strains. Colonies were then transferred into 30 mL BHIm or BHIs and incubated anaerobically at 37 °C. After 24 hr, 300 µL of each culture was transferred into a fresh 30 mL of BHIm or BHIs. After 18 h, these cultures were centrifuged (3000 x g for 15 min), resuspended in 1X PBS (HyClone, Logan, UT) to achieve a concentration of 1.0 × 10^9^ CFU/mL, and administered to mice via oral gavage (100 µL/mouse). For some experiments, *A. muciniphila* BAA-835 and *B. vulgatus* ATCC 8482 were prepared as described above and then mixed together in a 1:1 ratio prior to administering to mice (100 µL/mouse). A mixture containing both *A. muciniphila* YL44 and *B. vulgatus* RJ2H1 was also prepared for mice in this manner.

### Gnotobiotic mice with complex microbiomes

Germ-free C57BL/6 mice were born and reared in flexible film isolators and maintained under gnotobiotic conditions (temperature 20 °C, relative humidity 60%, 14 h light/10 h dark cycle) at the University of Nebraska-Lincoln. All mice were fed an autoclaved chow diet (LabDiet 5K67, Purina Foods, St. Louis, MO) ad libitum. The Institutional Animal Care and Use Committee at the University of Nebraska-Lincoln approved all procedures involving animals (protocols 1215 and 1700).

Four mouse microbiomes MFPL [36], MC608-F-a1 [18, 36], Wild116 [18, 36, 37], and BALBc.m3 [36, 38] were investigated as potential inocula for our experiments. MFPL refers to a C57BL/6 J mouse population of the Max F. Perutz Laboratories in Austria [36]). MC608-F-a1 refers to mice derived from a wild mouse population in the Massif Central region of France previously maintained at the Max Planck Institute for Evolutionary Biology (Plon, Germany; referred to as MC608-F-a1 in [36] and as A in [18]). Wild116 refers to wild mice caught in the United Kingdom [18, 36, 37]. BALBc.m3 refers to a BALB/c mouse population from a laboratory facility in the United Kingdom [36]. To produce standardized inocula of these microbiomes in sufficient amount for all experiments, female germ-free C57BL/6 mice were colonized with cecal contents from the donor mice and maintained under gnotobiotic conditions at the University of Nebraska-Lincoln for four weeks. Ceca were then collected, stored at −80 °C, and the contents resuspended (1:10 wt/vol) in reduced PBS as previously described [39] at the time of use.

DNA was extracted from the donor inoculum and recipient fecal samples, and microbial composition assessed by paired-end sequencing of the V5-V6 region of the 16 S rRNA gene using the MiSeq (Illumina) as previously described [18]. For taxa with a relative abundance greater than 0.03%, Bray-Curtis dissimilarities were calculated using the vegdist function in the R package vegan and then hierarchically clustered using the R function hclust set to the average agglomeration method. Data were then displayed in a heat map using packages ggplot and heatmap 2 (RStudio Team, Boston, MA). Our analysis revealed that the microbiomes of recipient mice clustered with their respective inocula (Fig. S1), suggesting overall successful stable engraftment. The analysis also showed only one of the four microbiomes (MFPL) contained sequences related to *A. muciniphila* and that the two microbiomes derived from wild mice (MC608-F-a1 and Wild116) lost the family *Bacteroidaceae*, which contains the genus *Bacteroides*, upon transplantation into germ-free mice. Cecal contents from the MFPL and MC608-F-a1 mice were pooled and selected as the positive and negative inocula in our study, respectively. The presence and absence of *A. muciniphila* and *B. vulgatus* in both microbiomes were confirmed using species-specific qPCR [38].

### Tests of persistence, coexistence, and the importance of colonization order

To determine to what degree the colonization dynamics of *A. muciniphila* and *B. vulgatus* strains were influenced by the presence or absence of the respective species present in the gut microbiome, female germ-free C57BL/6 mice were colonized with a microbiome identified as either positive (MFPL) or negative (MC608-F-a1) for *A. muciniphila* and *B. vulgatus* (week 0) as described above. Two weeks later (week 2), these mice were administered either a mixture of *A. muciniphila* BAA-835 and *B. vulgatus* 8482 or a mixture of *A. muciniphila* YL44 and *B. vulgatus* RJ2H1 for a total of two treatments. Each treatment consisted of five mice housed in one cage, and fecal samples were collected weekly at weeks 1, 2, 3, 4, 5, 6, and 7.

To test if colonization could be blocked or influenced by introducing one strain into the gut ecosystem before the other, female germ-free C57BL/6 mice were orally gavaged (week 0) with a negative microbiome (devoid of *A. muciniphila* and *B. vulgatus*) and a mixture of *A. muciniphila* BAA-835 and *B. vulgatus* 8482. Two weeks later (week 2), mice were orally gavaged with strains YL44 and RJ2H1. In a separate experiment, the importance of colonization order was tested by inverting the order of strain introduction. Specifically, GF mice were orally gavaged (week 0) with a negative microbiome and a mixture of either *A. muciniphila* YL44 and *B. vulgatus* RJ2H1. Two weeks later (week 2), mice were orally gavaged with strains BAA-835 and 8482. Studies were also performed to test the relative fitness of *A. muciniphila* and *B. vulgatus* strains when introduced at the same time. Female germ-free C57BL/6 mice were colonized with the negative microbiome at week 0 and then administered a mixture of all four strains at week 2. For all studies, each treatment consisted of five mice housed in one cage, and fecal samples were collected weekly at weeks 1, 2, 3, 4, 5, 6, and 7. See supplementary information for a description of methods used for *A. muciniphila* subtractive studies in mice.

### Quantitative real-time PCR (qPCR)

Strain-specific primers were designed to target unique genes not shared among the two test strains (identified using IMG-ER, Joint Genome Institute) [40]. Once unique genes were identified, potential specific primer pairs were generated using Prime3 software [41] and their quality assessed with NetPrimer (Premier Biosoft, San Francisco, CA). Primer specificity was verified bioinformatically by blasting primer sequences against a non-redundant DNA database for bacteria (NCBI) [42] and experimentally by qPCR using DNA isolated from the four strains (*B. vulgatus* RJ2H1, *B. vulgatus* 8482, *A. muciniphila* YL44, and *A. muciniphila* BAA-835) and the two microbiomes used in our experiments.

Strain-specific primers designed for this study were *A. muciniphila* BAA-835 forward CGGGGACAGTATATCGGGGA, reverse GAGATTCGGATAGCGCACCA; *A. muciniphila* YL44 forward GCCTTTCTTCAGCAAACGGG, reverse TCACAGCAGTTCAACAGGCA; *B. vulgatus* 8482 forward TCATCGTGGTCCATTGTCGG, reverse AACACCCCGTCAAAATTGCG; *B. vulgatus* RJ2H1 forward GCCGACGCTTTCTGACAAAA, reverse GAGGCGGCTTTCCATTGTTC. Thermocycling conditions for all four strain-specific primer pairs were: (i) initial denaturation step at 95 °C for 5 min; (ii) 35 cycles of 95 °C for 1 min, 64.2 °C for 30 sec, 72 °C for 30 sec; and (iii) one 20 min interval to generate a melting curve by progressively increasing the temperature from 60 °C to 95 °C.

Species-specific primers for *B. vulgatus* were also designed using a similar approach as for the strain-specific primers where primers targeted unique genes that were present in this species but absent in all other bacterial sequences reported in NCBI. The species-specific primers targeting *B. vulgatus* were forward GGCAGCATGGTCTTAGCTTGC, reverse GTGAACATGCGGACTCATGATG. Previously published species-specific primers were used to quantify *A. muciniphila* [43]. Thermocycling conditions for both *B. vulgatus* and *A. muciniphila* species-specific primer pairs were: (i) initial denaturation step at 95 °C for 5 min; (ii) 35 cycles of 95 °C for 1 min, 57 °C for 45 sec, and 72 °C for 45 sec; and (iii) one 20-min interval to generate a melting curve by progressively increasing the temperature from 60 °C to 95 °C.

All qPCRs were performed using SYBR green (Thermo Scientific, Lithuania) and a Mastercycler Realplex2 (Eppendorf AG, Hamburg, Germany). Specificity was tested using DNA from the strain of interest and the negative microbiomes utilized in this study. Optimal thermocycling conditions for qPCR were determined via gradient PCR using twelve temperatures between 53 °C and 63 °C (equal intervals) [44].

To make qPCR standard curves, aliquots of duplicate log-phase *A. muciniphila* or *B. vulgatus* cultures were plated on either BHIs or BHIm media for quantification of colony forming units per milliliter of culture (CFU/mL). A phenol chloroform method [45] was used for DNA extraction. Quantitative PCR including melt curve analysis, was performed on serially diluted (ten-fold) extracted DNA. Bacterial abundance was calculated based on the linear relationship between fluorescence of serially diluted DNA and corresponding CFU/mL [44]. Minimum limit of detection was established to be the lowest DNA dilution at which the relationship between CFU and fluorescence was linear.

### Whole metagenomic sequencing (WMS) and bioinformatic analyses

Metagenomic analysis of complex microbiomes was performed two weeks following transplant into germ-free mice but prior to the introduction of *A. muciniphila* and *B. vulgatus* test strains. Fecal DNA was extracted as described above [45] and sequenced at 10X depth on the MiSeq (Illumina) by Novogene America (Sacramento, CA). Quality check was performed with FastQC tools [46]. The script bbduk.sh (ktrim = r, k = 23, mink = 11, hdist = 1, Joint Genome Institute) was used to remove universal adaptors from samples. C57BL6/J (accession: GCA_000001635.9_GRCm39, NCBI) and *Homo sapiens* (accession: GCA_000001405.27_GRCh38, NCBI) genomes were utilized to remove mouse and human sequences, respectively, with bbsplit (BBmap tools, Joint Genome Institute). Each cleaned data set was mapped to either *B. vulgatus* 8482 (accession: GCA_000012825.1_ASM1282v1), *B. vulgatus* RJ2H1 (accession: GCA_002796815.1_ASM279681v1), *A. muciniphila* BAA-835 (accession: GCA_000020225.1) or *A. muciniphila* YL44 (accession: GCA_001688765.2) genomes from NCBI using BWA software set to default parameters [47]. SAMtools [47] was utilized to convert files into bam format and extract the mapped sequences. QualiMap [48] was used to determine the genome representation of the four test strains (8482, RJ2H1, BAA-835, and YL44) in each microbiome using the output “genome fraction coverage” of at least 1X. Results were plotted in Prism 8 (GraphPad Software, San Diego, CA). To determine genome representation of unique genomic sequences between *B. vulgatus* test strains in the positive microbiome, unique core sequences between the strains were extracted using RUCS [49] and verified using Mauve [50]. Mapping to determine and genome representation of strain specific genomic sequences within the positive microbiome was then performed with BWA and QualiMap as described above.

Metagenome de novo sequence assembly was performed with MEGAHIT and evaluated by mapping the original reads to the assembled sequences using BWA software and SAMtools as described above. The software MetaBAT 2 [51] set to default parameters for complex communities was used to collect contigs predicted to the same species into a single bin. Taxonomic identification of bins belonging to *B. vulgatus* and *A. muciniphila* was performed by Blastn with 5.3 × 10^6^ and 2.8 × 10^6^ base pairs for *B. vulgatus A. muciniphila* related bins, respectively, which was similar to the average genome sizes reported for these species (NCBI database) [52, 53]. Genomic representation of test strains against resident *B. vulgatus* and *A. muciniphila* contigs was performed with BWA, SAMtools, and Qualimap as described above. Taxonomic profiles of the metagenomic dataset were generated using MetaPhlAn3 (v3.0.2) [54] with default settings. Taxa presenting in no less than three mice in at least one treatment were retained for data visualization using GraPhlAn (v1.1.3) [55] and the R package pheatmap.

### Genomic comparisons between test strains

IMG/MER tools [40, 42] from the Joint Genome Institute were used to calculate genome-wide average nucleotide identity (gANI) and alignment fractions (AF) for *A. muciniphila* BAA-835 versus YL44 and *B. vulgatus* 8482 versus RJ2H1 as previously described [56]. Genome size information from strains reported at NCBI was used to generate histogram distribution using the ggplot2 package in RStudio (RStudio Team, Boston, MA). The IMG/MER phylogenetic profiler tool was used to perform gene context analysis and identify unique genes and protein families (Pfam) for each strain [40, 42, 57]. Classifications of identified protein families were made based on descriptions in the protein family database [58] and the Universal Protein Resource [59]. Pie charts and stacked bar graphs were create using Prism 8 (GraphPad Software, San Diego, CA).

### Quantification and statistical analysis

All longitudinal data were analyzed using two-way ANOVA repeated measures and Tukey test multiple pairwise comparisons using Prism 8 (GraphPad Software). A *p*-value of 0.05 was considered significant.

## Results

### *A. muciniphila* and *B. vulgatus* strains only colonized gnotobiotic mice harboring complex microbiomes devoid of these species

To confirm published studies showing that bacterial colonization in the mammalian gut depends on the absence of related bacteria [17, 18, 27, 60], we tested the persistence of *A. muciniphila* and *B. vulgatus* strains in mice harboring complex microbiomes with (positive) and without (negative) these species. We established a microbiome in germ-free mice that contained these species (MFPL) as well as a microbiome that lacked the phylum Verrucomicrobia and the family *Bacteroidaceae* (MC608-F-a1). After two weeks of colonization, we confirmed the presence/absence of *A. muciniphila* and *B. vulgatus* by species-specific qPCR. WMS verified the presence of *A. muciniphila* and *B. vulgatus* in the positive microbiome and the absence of these taxa (along with their respective phyla and families) in the negative microbiome (Fig. S2a–c). Approximately 85% of the genome sequences from test strains *A. muciniphila* BAA-835 and YL44 and *B. vulgatus* 8482 and RJ2H1 were represented in the metagenomes of the positive microbiome (Fig. S2d). In addition, ~70–80% of the genome sequences from the experimental *A. muciniphila* and *B. vulgatus* strains was shared with the resident *B. vulgatus* and *A. muciniphila* populations present in the positive microbiome (Fig. S2e, f). In contrast, the genomes of all test strains were poorly represented in the negative microbiome (Fig. S2e, f).

**Fig. 1:**
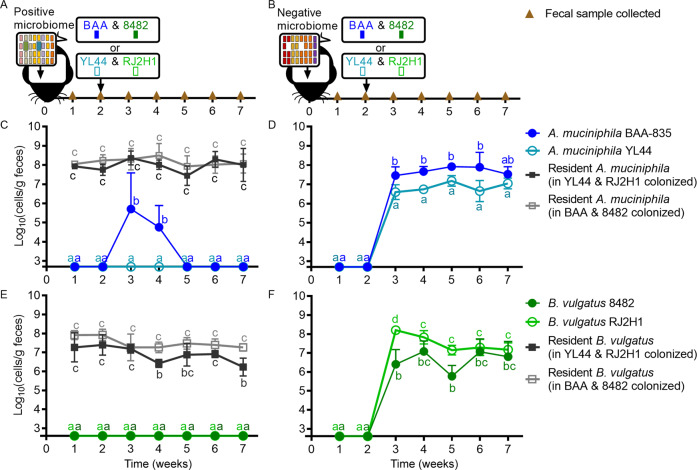
*A. muciniphila* and *B. vulgatus* strains only colonized gnotobiotic mice harboring complex microbiomes devoid of these species. Experimental design to test colonization of strains in mice harboring a microbiome with (positive; **A**) and without (negative; **B**) *A. muciniphila* and *B. vulgatus*. Brown triangles represent timepoints for fecal sample collections. Black arrows represent colonization events with microbiomes or *A. muciniphila* and *B. vulgatus* strains. Week 2 fecal samples were collected prior to inoculating with test strains. Abundance of *A. muciniphila* species (gray), strain BAA-835 (dark blue), and strain YL44 (light blue) in mice harboring either a positive (**C**) or a negative (**D**) microbiome. Abundance of *B. vulgatus* species (gray), strain 8482 (dark green), and strain RJ2H1 (light green) in mice harboring either a positive (**E**) or a negative (**F**) microbiome. Values are presented as mean ± the standard deviation. Time points with different letters are significantly (*p* < 0.05) different from one another at indicated timepoints by two-way ANOVA repeated measures and Tukey test multiple pairwise comparisons in each plot.

We then orally administered experimental strains of *A. muciniphila* and *B. vulgatus* to mice harboring either a positive or negative microbiome. Using strain-specific qPCR (Fig. 1A), we found that neither the *A. muciniphila* nor the *B. vulgatus* test strains colonized mice carrying a microbiome that contained these species (Fig. 1C, D). In contrast, stable persistence of test strains was achieved in mice harboring the negative microbiome that was naturally devoid of *A. muciniphila* and *B. vulgatus* (Fig. 1D, F). These results suggest that colonization of exogenous bacterial strains can occur in the absence, but not presence, of the same species (or species representing the genetic potential of the incoming strains) in the resident microbiome, thus confirming previous findings in both mice and humans [17, 18].

### Colonization of *A. muciniphila*, but not *B. vulgatus*, was strictly governed by competitive exclusion, while priority effects were detectable for both species

**Fig. 2:**
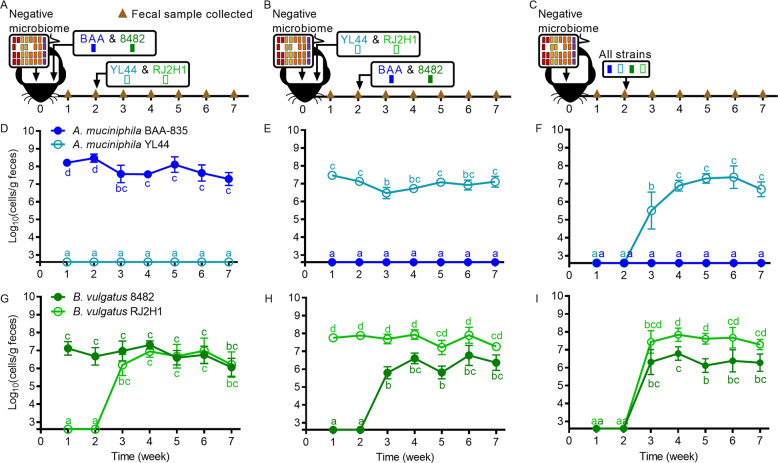
Colonization of *A. muciniphila*, but not *B. vulgatus*, was strictly governed by competitive exclusion, while priority effects were detectable for both species. All mice were colonized at week 0 with a negative microbiome devoid of *A. muciniphila* and *B. vulgatus*. Competition experiments with strains of *A. muciniphila* and *B. vulgatus* were performed as follows: (**A**, **D**, **G**) *A. muciniphila* BAA-835 and *B. vulgatus* 8482 were introduced at week 0 prior to introduction of *A. muciniphila* YL44 and *B. vulgatus* RJ2H1 at week 2. **B**, **E**, **H** *A. muciniphila* YL44 and *B. vulgatus* RJ2H1 were introduced at week 0 prior to introduction of *A. muciniphila* BAA-835 and *B. vulgatus* 8482 at week 2. **C**, **F**, **I** *A. muciniphila* and *B. vulgatus* strains were all introduced into mice at the same time. **D**–**F** Abundance of *A. muciniphila* strain BAA-835 (dark blue) and strain YL44 (light blue) in feces. **G**–**I** Abundance of *B. vulgatus* strain 8482 (dark green) and strain RJ2H1 (light green) in feces. Week 2 fecal samples were collected prior to inoculating with test strains. Values are presented as mean ± the standard deviation. Time points with different letters are significantly (*p* < 0.05) different from one another by two-way ANOVA repeated measures and Tukey test multiple pairwise comparisons among treatments of *A. muciniphila* (**D**–**F**) or *B. vulgatus* (**G**–**I**).

Although the findings above suggest that concepts such as competitive exclusion or limiting similarity pertain to gut ecosystems [61], they do not provide direct evidence because the differences between the positive and negative microbiomes pertained not only to the target species but also to their respective phyla and families. Our next experiments therefore aimed to specifically evaluate the outcome of strain-to-strain competition and test whether it resulted in competitive exclusion. We first colonized germ-free mice with a “permissive” negative microbiome devoid of *A. muciniphila* and *B. vulgatus* and a mixture of both *A. muciniphila* BAA-835 and *B. vulgatus* 8482. Two weeks later, mice were colonized with a mixture of both *A. muciniphila* YL44 and *B. vulgatus* RJ2H1 (Fig. 2A). A second cohort of mice harboring permissive microbiomes were colonized with both *A. muciniphila* YL44 and *B. vulgatus* RJ2H1 first, followed by *A. muciniphila* BAA-835 and *B. vulgatus* 8482 two weeks later (Fig. 2B). Finally, a third cohort of mice colonized with the permissive microbiome was used to test all four strains together to determine fitness differences of the strains (Fig. 2C).

For *A. muciniphila*, we observed that the strain arriving first stably colonized, while the second was only temporarily detected, indicating competitive exclusion (Fig. 2D, E). Notably, competition outcomes were strictly dependent on arrival order, with the first colonizer excluding the later colonizer, thus demonstrating that priority effects are of paramount importance. Priority effects were strong enough to abrogate the fitness differences observed between the two *A. muciniphila* strains. Strain BAA-835 outcompeted YL44 when it colonized first, although it was outcompeted by YL44 when both strains were inoculated together (Fig. 2F).

In contrast to the findings obtained for *A. muciniphila*, both strains of *B. vulgatus* were able to stably colonize independently of strain arrival succession (Fig. 2G–I). Despite stable coexistence, the two strains still influenced one another’s abundance, indicating competitive interactions that were further influenced by priority effects. Although the sum total abundance of the two *B. vulgatus* strains was equivalent across all colonization scenarios (Fig. S3), the maximum abundance levels for individual strains were significantly higher (mean estimate of difference 0.5–1.0 log, *p* < 0.05) when they colonized first as compared to when colonizing second (Fig. 2G vs. H). Second, the higher population level of strain RJ2H1 (~10^8^ cells/g feces) compared to 8482 (~10^6^ cells/g feces) observed when RJ2H1 colonized first or at the same time as 8482 was no longer detectable when strain 8482 was introduced first (both strains at ~10^7^ cells/g feces). These findings indicate that although the two *B. vulgatus* strains coexisted and were not subjected to strict competitive exclusion, the two strains still affected each other’s population levels and priority effects clearly influenced competition outcomes.

### Strain-to-strain differences in traits enabling niche differentiation may explain the distinct competition outcomes between *A. muciniphila* and *B. vulgatus* strains

**Fig. 3:**
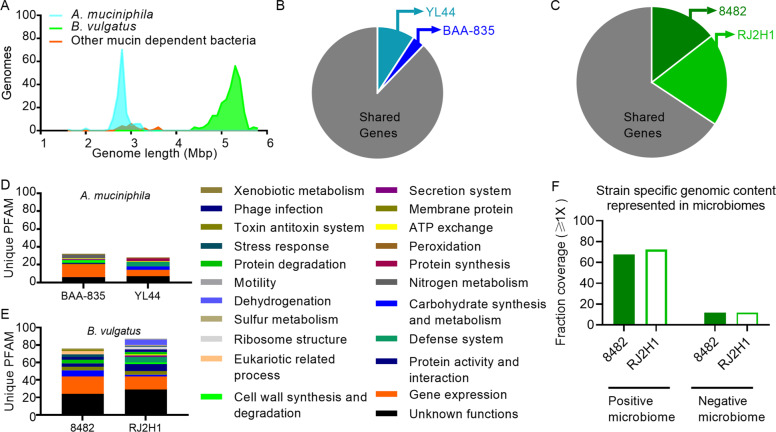
Genomic differences between *A. muciniphila* and *B. vulgatus* strains. **A** Distribution of bacterial genome sizes. Mbp = Millions of base pairs of total DNA sequence length for each strain in the NCBI genome database. **B** Shared (gray) and unique genes for *A. muciniphila* BAA-835 (dark blue) and YL44 (light blue) in direct genome comparisons. **C** Shared (gray) and unique genes for *B. vulgatus* 8482 (dark green) and RJ2H1 (light green). Sizes of pie charts are scaled to represent proportional differences between *A. muciniphila* and *B. vulgatus* genomes. Biological processes related to unique protein families are depicted for *A. muciniphila* (**D**) and *B. vulgatus* (**E**) strains. Each protein is grouped by colors that represent an individual biological process. **F** Fraction of the genome sequences unique to *B. vulgatus* 8482 or RJ2H1 (in genomic comparisons between the two strains) that is represented in the positive and negative (permissive) pretreatment microbiomes.

Similarities between bacteria increase competition for resources and may result in competitive exclusion [62]. However, differences in resource requirements can lead to resource partitioning and niche differentiation and thus constitute a stabilizing mechanism that increases the chance of coexistence [25]. We therefore sought to investigate whether genetic relationships could explain the differences in coexistence patterns observed between *A. muciniphila* (strict competitive exclusion) and *B. vulgatus* (coexistence with competitive interactions) strains. An assessment of genetic relatedness using genome-wide average nucleotide identity (gANI) and alignment fraction (AF) metrics [56] revealed that the genomes of the two *A. muciniphila* strains were more similar to one another than those of the *B. vulgatus* strains (gANI values of 99.10 vs. 98.68 and AF values of 93.37 vs. 75.58 for *A. muciniphila* and *B. vulgatus*, respectively; Table 1). We also observed that the genomes of *A. muciniphila* strains were smaller than those of *B. vulgatus* strains (Fig. 3A) and consistent in size with organisms exhibiting specialist behaviors [63], such as the mucin degradation/utilization for which *A. muciniphila* is highly specialized [64]. Each *A. muciniphila* strain differed from one another in only a few unique encoded proteins related to processes of gene regulation and nitrogen metabolism (Fig. 3B, D). In contrast, the *B. vulgatus* strains differed by multiple proteins related to processes of gene regulation, carbohydrate binding and metabolism, phage infection, stress responses, and protein degradation (Fig. 3C, E).

**Table 1:** Genome-wide average nucleotide identities among the strains utilized in this study.

Genome1 Name	Genome2 Name	ANI1- > 2	ANI2- > 1	AF1- > 2	AF2- > 1
*Bacteroides vulgatus* ATCC 8482	*Bacteroides vulgatus* RJ2H1	98.7	98.7	75.6	75.6
*Bacteroides vulgatus* ATCC 8482	*Akkermansia muciniphila* YL44	65.5	65.7	1.0	1.9
*Akkermansia muciniphila* ATCC BAA-835	*Akkermansia muciniphila* YL44	99.1	99.1	93.4	90.7
*Akkermansia muciniphila* ATCC BAA-835	*Bacteroides vulgatus* RJ2H1	65.7	65.7	2.0	1.1

Average nucleotide identity (ANI) and Alignment Fraction (AF) are measuring genetic relatedness. ANI1 and AF1 are calculated with Genome 1 as the reference. ANI2 and AF2 are calculated with Genome 2 as the reference.

Consistent with these observations, *B. vulgatus* strains 8482 and RJ2H1 differed in terms of the carbohydrate substrates they utilized in vitro (Table S1). Specifically, strain 8482 exhibited superior growth on N-acetyl glucosamine and chondroitin sulfate as well as on simple sugars such as fructose and glucose, whereas strain RJ2H1 grew well on several starches, including amylopectin, glycogen, pullulan, and rhamnogalacturonan I, and xylan.

Altogether, these findings suggest that competitive exclusion between *A. muciniphila* strains is likely related to high genetic similarity. In contrast, the coexistence of *B. vulgatus* strains may be determined by unique genes and patterns of carbohydrate metabolism whereby strain 8482 specializes towards host-derived carbohydrates and simple dietary sugars while strain RJ2H1 specializes on complex dietary substrates to promote niche differentiation. Importantly, ~70% of the genome sequences found to be unique to either *B. vulgatus* 8482 or RJ2H1 in direct genome comparisons between these strains, which may encode the traits that enable niche partitioning, was represented in the positive microbiome (Fig. 3f), thus providing an explanation for why this microbiome excluded both *B. vulgatus* strains (Fig. 1E).

### A subtractive antibiotic strategy enabled replacement of an established *A. muciniphila* strain

Our observations for *A. muciniphila* suggest that the establishment of a new strain of this species is prevented via competitive exclusion by a resident strain of the same species whose fitness is enhanced through priority effects. This finding provides a mechanism underlying the difficulty in establishing new strains within the gut microbiota and implies that successful colonization of novel *A. muciniphila* strains depends upon the reduction or removal of a pre-existing, related strain within the microbiome using a subtractive approach [15]. One potential strategy for such modulation is antibiotic treatment [65]. We therefore hypothesized that an antibiotic regimen would allow us to replace an established strain of *A. muciniphila* with a novel strain. We selected candidate antibiotics based on Derrien et al., who reported that *A. muciniphila* was susceptible to ampicillin (AMP) [64] and other reports describing antibiotics with effects on *A. muciniphila* or Verrucomicrobia [31, 66, 67]. Candidate antibiotics were first screened in vitro for their ability to attenuate growth of *A. muciniphila* BAA-835 and YL44 (Table S2). Macrolide tylosin tartrate (MTT), clarithromycin (CLA), and AMP all limited the growth of BAA and YL44 in vitro.

**Fig. 4:**
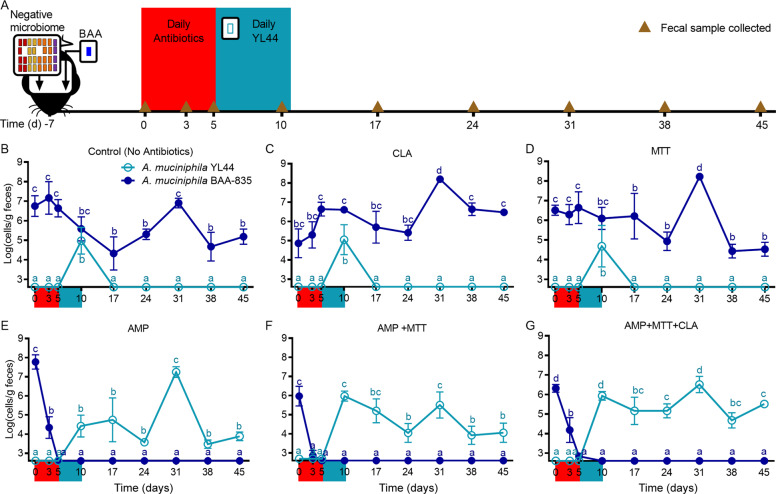
A subtractive antibiotic strategy enabled replacement of an established *A. muciniphila* strain. **A** Experimental design depicting conventionalization of germ-free mice with a negative microbiota devoid of *A. muciniphila* and colonization with strain BAA-835 at day −7. On day 0, mice were treated with antibiotics or regular drinking water for five days. On day 5, mice receiving antibiotics were returned to regular drinking water and fecal samples were collected. Also on day 5, mice began receiving daily gavages of YL44 for five days. Brown triangles represent timepoints for fecal sample collections. Abundance of *A. muciniphila* BAA-835 (dark blue) and *A. muciniphila* YL44 (light blue) in feces of mice not treated with antibiotics (**B**) or treated with CLA alone (**C**), **D** MTT alone, **E** AMP alone, **F** AMP + MTT, **G** AMP + MTT + CLA. Values are presented as mean ± the standard deviation. In each plot, time points with different letters are significantly (*p* < 0.05) different from one another by two-way ANOVA repeated measures and Tukey test multiple pairwise comparisons.

To systematically test the efficacy of an antibiotic-based subtractive strategy, germ-free mice were first conventionalized with a permissive microbiota devoid of *A. muciniphila* and then colonized one week later with strain BAA-835. One week after the addition of BAA-835, mice received either CLA, MTT, AMP, AMP + MTT, AMP + MTT + CLA, or no antibiotics in their drinking water for five days. On the fifth day, mice receiving antibiotics were returned to regular drinking water and began receiving daily gavages of YL44 for five days (Fig. 4A). In agreement with findings from our previous experiment (Fig. 2d), YL44 could not colonize control mice and did not influence BAA-835 population levels (Fig. 4B). Treatment with either MTT or CLA resulted in the same outcomes as observed for control mice (Fig. 4C and D). In contrast, administering AMP alone or in combination with MTT and/or CLA depleted the existing BAA strain to undetectable levels and enabled stable colonization of YL44 for the duration of the five-week experiment (Fig. 4E–G). Together, these results demonstrate that subtractive antibiotic treatment can be used as a strategy to successfully remove a pre-existing *A. muciniphila* strain from an assembled gut microbial community and replace it with a novel strain that would otherwise be excluded by competitive exclusion. These experiments also further confirm that the competitive interactions between *A. muciniphila* strains detected in previous experiments are not due to interactions with *B. vulgatus*.

## Discussion

Recent studies suggest that colonization of an incoming microbe in a microbiome is determined by the presence of closely related inhabitants [17, 18, 27, 60], thus suggesting that principles such as competitive exclusion apply to gut ecosystems. However, the applicability of these concepts has not been empirically established, nor have there been attempts to apply them to exchange strains in an assembled community. Using systematic experiments in gnotobiotic mice, we found that *A. muciniphila* strains, which have narrow and likely overlapping niches, excluded one another from the microbiome whereas *B. vulgatus* strains could coexist, likely through niche partitioning, but still showed ecological interactions pointing to competition. We observed that competitive interactions between both *A. muciniphila* and *B. vulgatus* strains were influenced by time of arrival, thus establishing the importance of priority effects as a determinant of coexistence. Finally, we demonstrated that antibiotic treatments can be used to replace *A. muciniphila* strains within an assembled microbiome. Altogether, our results suggest that important aspects of coexistence theory (e.g., the ability to partition niches and the impact of priority effects on fitness differences) determine strain competition outcomes in gut ecosystems and suggest that such principles can be applied to design strategies that modulate microbiomes.

According to modern coexistence theory, coexistence in an ecosystem is determined by the degree to which members differ in fitness (i.e., equalizing mechanisms), their niches (i.e., stabilizing mechanisms), and their time of arrival [25, 26, 68, 69]. Our findings agree with these principles, which can provide an explanation for the profound differences between *A. muciniphila* and *B. vulgatus* with respect to coexistence. *A. muciniphila* colonizes the mucus layer [29], an anatomically-defined structure composed primarily of mucin agglomerates [70], and preferentially metabolizes mucin over other carbohydrates [64, 71], thus restricting the variability of resources in its ecological niche. These behaviors are also consistent with the small genomic differences between strains BAA-835 and YL44, indicating that these strains are ecologically very similar. Consequently, one would predict that stabilizing mechanisms are reduced between competing *A. muciniphila* strains because they are unable to partition niches, which should result in strict competitive exclusion where the better competitor excludes the other strain. These assumptions are supported by our current observations for *A. muciniphila*. However, our findings indicate that the ability of these strains to compete (i.e., fitness) is influenced by arrival order. Specifically, the less fit *A. muciniphila* strain won the competition when it arrived first. Our results therefore confirm the importance of priority effects in gut ecosystems [18, 26] and demonstrate that these effects can be strong enough to overcome inherent fitness differences between *A. muciniphila* strains.

We were surprised to not see a competitive advantage for the *A. muciniphila* strain isolated from mice (YL44) over the strain of human origin (BAA-835) given that host adaptation has been shown for other bacteria closely associated with host epithelia (e.g., *Limosilactobacillus reuteri*) [72, 73]. It therefore appears that host origin plays no role in influencing the competitive abilities of *A. muciniphila*. However, one should consider that the microbiomes of mice housed in modern research vivaria are often highly artificial [74], and the microbiome derived from wild mice in our study did not contain *A. muciniphila*. We therefore screened the literature to determine if *A. muciniphila* is truly of murine origin. Results from several studies confirmed our observation that *A. muciniphila* has not been detected in the microbiomes of wild mice [75, 76]. Overall, these findings suggest that *A. muciniphila* YL44 shares no evolutionary relationship with mice, thus explaining why it does not exhibit a stronger ecological performance when compared with a human isolate.

Unlike *A. muciniphila* strains, *B. vulgatus* strains do not exclude each other. This observation can be explained by the existence of stabilizing mechanisms that lead to niche differentiation. In contrast to *A. muciniphila*, whose niche is restricted to the mucus layer, *B. vulgatus* colonizes food particles and scattered luminal regions adjacent to the colonic mucosa [77, 78]. The *B. vulgatus* niche is therefore likely much more complex and dynamic than that of *A. muciniphila* and provides a larger range of substrates that become accessible by different strains. Indeed, the larger genomes for *B. vulgatus* compared to *A. muciniphila* suggest that these strains are generalists [14]. Consistently, *B. vulgatus* strains 8482 and RJ2H1 also differed in diversity of carbohydrate and protein degradation capabilities and substrate binding/utilization, which suggests that they are able to partition niches and stably coexist even if equalizing mechanisms are low due to fitness differences [25]. Despite the stable coexistence of *B. vulgatus* isolates, we still detected competitive interactions that were influenced by priority effects. *B. vulgatus* strains achieved a higher colonization level when they arrived first, and fitness differences could be overcome through early arrival. Overall, our observations for *B. vulgatus* strains are consistent with coexistence theory in that strains capable of partitioning niches can coexist. However, our experiments have not identified niche differentiation within *B. vulgatus* on a mechanistic level, nor did they assess competitive interactions among closely related species (as mice harboring the negative microbiome lacked *Bacteroidaceae*) or how such interactions affect the niche of *B. vulgatus*. Future studies to experimentally determine the mechanisms of niche partitioning within *B. vulgatus* in the context of complex communities are therefore required. Regardless of the exact mechanisms, a novel and important finding for our understanding of the gut ecosystem is that even if strains stably coexist, their population levels are still affected by competition and influenced by priority effects.

Taken together, our findings suggest that key aspects of modern coexistence theory can be applied to understand fundamental characteristics of gut microbial communities. In accordance with that theory, species and strains with similar fitness levels can coexist when niche differences are sufficiently large to reduce overlap in resource usage [25]. In contrast, if niches overlap too much, then fitness differences prevent coexistence and result in competitive exclusion [25]. Our observations for *A. muciniphila* provide experimental evidence to support previous findings that point to competitive exclusion as a mechanism for colonization resistance [17, 18, 27, 60]. Most importantly, our findings agree with observations for strain coexistence in humans [75, 79, 80]. In particular, Truong et al. showed that most individuals harbor only one strain of *A. muciniphila* but tend to carry multiple resident *B. vulgatus* strains [80], suggesting that the ecological principles underpinning strain coexistence in mice may also apply to humans.

Of particular significance is our demonstration that priority effects can be strong enough to modify and even reverse fitness differences between strains and consequently alter competition outcomes, regardless of whether they result in strict competitive exclusion or coexistence. By demonstrating that arrival order alters fitness differences among strains and ultimately reverses the outcome of their competition, our findings extend previous research [18] describing that priority effects can influence the persistence of individual colonizers and the historical contingency of microbiome assembly. Priority effects, in the context of modern coexistence theory, can therefore provide mechanistic explanations for key characteristics of gut ecosystems. First, competitive exclusion, in combination with priority effects, provides a mechanism that enhances colonization resistance of gut microbiomes as it endows the established organism with a fitness advantage over later arrivals, thereby providing an explanation for microbiome stability, resistance, and resilience [81, 82]. Second, priority effects could explain why maternal-derived bacteria, which are likely to arrive early, are more stable colonizers compared to non-maternal strains [83, 84]. Third, priority effects can influence the abundance of coexisting community members, thus offering a mechanism by which arrival order (which is likely to be largely stochastic) creates differences in gut microbiota composition and explains, in part, the substantial interindividual variation observed in gut microbiomes [18]. We acknowledge that we have not established the mechanisms by which priority effects emerge. Such effects may manifest through ecological (e.g., niche pre-emption) or evolutionary mechanisms (e.g., by early colonizers adapting to the host, thereby becoming more competitive), but could also be influenced by the host immune system if early colonizing strains induce immune responses that benefit their persistence. Future studies should focus on elucidating these mechanisms.

Considering these ecological concepts, there are substantial practical implications for successfully introducing a new strain into the gut microbiota: (i) closely-related established strains must be absent [17, 38]; (ii) the incoming strain, which is disadvantaged by priority effects, must outcompete the resident strain; or (iii) the resident strain currently occupying the niche must first be removed through subtractive approaches. The third strategy has been both proposed and applied in some fecal microbiota transplant (FMT) studies [17, 85, 86], and a recent meta-analysis of newly generated and available metagenomes from post-FMT patient samples revealed that antibiotic pretreatment enhanced donor strain engraftment [19]. Our findings provide a mechanistic foundation for the effect of antibiotics on strain engraftment by demonstrating that a subtractive approach based on antibiotic treatment could indeed be used to replace an established *A. muciniphila* strain with a new one. Specifically, administration of antibiotics suppressed the abundance of the competing, early-arriving *A. muciniphila* strain and opened a niche for the late-arriving strain to colonize. Subtractive approaches such as antibiotics may therefore enable microbiome modulation by both suppressing competing strains and opening niches for new colonizers. We acknowledge that the use of broad-spectrum antibiotics such as ampicillin to replace strains has substantial disadvantages for translation into humans. More targeted subtractive methods, including the use of bacteriophages [87] or CRISPR/cas systems [88], are likely more desirable, and, if applied in agreement with ecological theory, could pave the way for precision tools to modulate microbiomes. Importantly, our study provides the proof-of-concept that such approaches can be successful.

In conclusion, this study demonstrates the applicability of the central aspects of modern coexistence theory to gut ecosystems and that such theory can be used to understand engraftment of incoming microbes. Our findings provide potential explanations for many fundamental characteristics of the gut microbiome, including stability, colonization resistance, enhanced stability of maternally-acquired strains, and drivers of inter-subject variation. Furthermore, this work informs future intervention studies aimed at modulating gut ecosystems using live microbes, which will likely need to be personalized based on an individual’s baseline microbiome and attempt to remove competitors through subtractive approaches. Admittedly, our study only tested two strains each of two important bacterial species, and the ecological principles governing other microbiota members and the context in which they apply, might differ. Future research should extend our work to include additional strains with known niche characteristics, evaluate their competition in the context of the wider microbial community, and focus efforts on the exact mechanisms by which strains coexist or compete and priority effects emerge. Additionally, gnotobiotic mouse models could be designed to facilitate the investigation of interspecies interactions at various taxonomic levels (e.g., including other *Bacteroides* species with well-characterized niches [35]). Studies could also be performed that combine gnotobiotic models of disease with genetic mutants to determine the bacterial traits that contribute to niche partitioning to further understand the exact mechanisms by which microbes coexist in gut ecosystems and how their relationships are influenced by pathologies that alter the gut microbiota.

## Supplementary information

Supplemental Material

## Data Availability

The datasets generated during the current study are available in Mendeley with the identifier 10.17632/gbc76stz42.2. The metagenomic data that support the findings of this study have been deposited in the NCBI Sequence Read Archive under the BioProject ID PRJNA788901.
