# Correction: Experimental evaluation of ecological principles to understand and modulate the outcome of bacterial strain competition in gut microbiomes

**DOI:** 10.1038/s41396-022-01227-6

**Published:** 2022-04-20

**Authors:** Rafael R. Segura Munoz, Sara Mantz, Ines Martínez, Fuyong Li, Robert J. Schmaltz, Nicholas A. Pudlo, Karthik Urs, Eric C. Martens, Jens Walter, Amanda E. Ramer-Tait

**Affiliations:** 1 Department of Food Science and Technology, University of Nebraska-Lincoln, Lincoln, NE USA; 2 Nebraska Food for Health Center, University of Nebraska-Lincoln, Lincoln, NE USA; 3 Department of Agricultural, Food and Nutritional Science, University of Alberta, Edmonton, AB Canada; 4 Department of Biological Sciences, University of Alberta, Edmonton, AB Canada; 5 Department of Infectious Diseases and Public Health, Jockey Club College of Veterinary Medicine and Life Sciences, City University of Hong Kong, Kowloon, Hong Kong SAR China; 6 Department of Microbiology and Immunology, University of Michigan Medical School, Ann Arbor, MI USA; 7 APC Microbiome Ireland, School of Microbiology, and Department of Medicine, University College Cork, Cork, Ireland

**Keywords:** Microbial ecology, Microbiome

Correction to: *The ISME Journal* 10.1038/s41396-022-01208-9, published online 24 February 2022

Following online publication of this article, the authors noted an error in Fig. 2a. The box above the 2-week timepoint should have shown strains YL44 & RJ2H1 and not strains BAA & 8482. The corrected figure should have appeared as shown below:
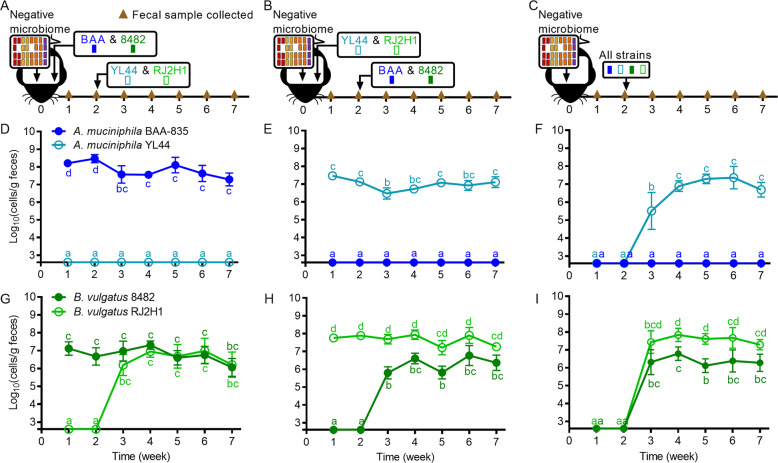


The original article has been corrected.

