# Supplementary material for: Experimental evaluation of ecological principles to understand and modulate the outcome of bacterial strain competition in gut microbiomes
Source: ISME J. 2022 Feb 24;16(6):1594–604. doi: 10.1038/s41396-022-01208-9 (PMC9122919; doi:10.1038/s41396-022-01208-9)
Supplement: Supplementary file 1 — Supplemental Material [file 41396_2022_1208_MOESM1_ESM.pdf]

Supplementary Information

Experimental evaluation of ecological principles to understand and modulate the outcome of bacterial strain competition in gut microbiomes

Rafael R. Segura Munoz[1,2], Sara Mantz[1], Ines Martínez[3,4], Fuyong Li[3,5], Robert J. Schmaltz[1], Nicholas A. Pudlo[6], Karthik Urs[6], Eric C. Martens[6], Jens Walter [3,4,7*], and Amanda E. Ramer-Tait[1,2,*]

[1]Department of Food Science and Technology, University of Nebraska-Lincoln, USA; [2]Nebraska Food for Health Center, University of Nebraska-Lincoln, USA; [3]Department of Agricultural, Food and Nutritional Science, University of Alberta, Edmonton, Canada; [4]Department of Biological Sciences, University of Alberta, Edmonton, Canada; [5]Department of Infectious Diseases and Public Health, Jockey Club College of Veterinary Medicine and Life Sciences, City University of Hong Kong, Kowloon, Hong Kong SAR, China; [6]Department of Microbiology and Immunology, University of Michigan Medical School, USA, [7]APC Microbiome Ireland, School of Microbiology and Department of Medicine, University College Cork-National University of Ireland, Cork, Ireland.

* Correspondence:

Amanda E. Ramer-Tait, Department of Food Science and Technology, University of Nebraska-Lincoln, 1901 N 21st St., 260 Food Innovation Center, Lincoln, NE 68588. E-mail: aramer-tait2@unl.edu. Telephone: +1-402-472-7293

Jens Walter, APC Microbiome Ireland, School of Microbiology, and Department of Medicine, University College Cork – National University of Ireland, 4.05 Biosciences Building, Cork, T12 YT20, Ireland. Email: jenswalter@ucc.ie. Phone: +353 (0)21 490 1773

Supporting Methods

Tests for antibiotic sensitivity

To determine sensitivity of *Akkermansia* strains to antibiotics *in vitro*, BHIm agar plates containing multiple combinations of ampicillin (AMP), microlide Tylosine tartrate (MTT), and clarithromycin (CLA) were prepared. A set of BHIm and BHIs agar plates prepared without antibiotics were used as a control. A 500 µL aliquot of each strain was spread to create a lawn; plates were then incubated as described above and monitored for the presence/absence of CFUs. The sensitivity of *A. muciniphila* strains to additional antibiotics (Table S1) was evaluated via disk diffusion tests using a second set of BHIm and BHIs agar plates prepared without antibiotics [1].

Subtractive microbiome modulation in mice using antibiotics

Germ-free mice were gavaged with the negative microbiome and *A. muciniphila* BAA-835 (day -7). One week later (day 0), mice were either left untreated (control) or given one of the following five antibiotic treatments for five days: MTT, CLA, AMP, AMP + MTT, or AMP + MTT + CLA. Antibiotic treatments containing AMP and/or MTT were administered via the drinking water at 1.33 mg/mL and 1 mg/mL, respectively [2]. Antibiotics in the drinking water were replaced daily during the five-day treatment period. Because CLA has low solubility in water, CLA was suspended at 1 mg/mL in a 1:5 solution of dimethyl sulfoxide (Sigma-Aldrich) and water [3] and then orally gavaged daily to mice for five days (100 uL/mouse) [3]. Cages were changed daily during the antibiotic treatment period to limit recolonization through coprophagy. Each treatment consisted of three to six mice housed two to five per cage. Fecal samples were collected prior to the introduction of antibiotics (day 0) and on days 3 and 5 during antibiotic treatment. On day 5 (after the fecal collection), all antibiotic solutions were replaced

with regular drinking water. Mice were orally gavaged with *A. muciniphila* YL44 (3 x 10^8 CFU/mL) daily for five days, and fecal samples were collected on day 10 after last YL44 gavage and then weekly for 5 weeks.

Data availability

Raw data for this manuscript can be found at Mendeley: https://data.mendeley.com/v1/datasets/gbc76stz42/draft?a=e350f137-9e36-4ded-bf38-83bd9ead4644. Availability of 16S rRNA and metagenomic datasets is in-progress.

Supporting References

1. Brown DF, Kothari D. Comparison of antibiotic discs from different sources. J Clin Pathol. 1975;28:779-783.
2. Livanos AE, Greiner TU, Vangay P, Pathmasiri W, Stewart D, McRitchie S *et al*. Antibiotic-mediated gut microbiome perturbation accelerates development of type 1 diabetes in mice. Nat Microbiol. 2016;1:16140.
3. Tessier PR, Kim MK, Zhou W, Xuan D, Li C, Ye M *et al*. Pharmacodynamic assessment of clarithromycin in a murine model of pneumococcal pneumonia. Antimicrob Agents Chemother. 2002;46:1425-1434.
4. Lagkouvardos I, Pukall R, Abt B, Foesel BU, Meier-Kolthoff JP, Kumar N *et al*. The Mouse Intestinal Bacterial Collection (miBC) provides host-specific insight into cultured diversity and functional potential of the gut microbiota. Nat Microbiol. 2016;1:16131.
5. Martinez I, Maldonado-Gomez MX, Gomes-Neto JC, Kittana H, Ding H, Schmaltz R *et al*. Experimental evaluation of the importance of colonization history in early-life gut microbiota assembly. Elife. 2018;7.
6. Weldon L, Abolins S, Lenzi L, Bourne C, Riley EM, Viney M. The gut microbiota of wild mice. PLoS One. 2015;10:e0134643.
7. Segura Munoz RR, Quach T, Gomes-Neto JC, Xian Y, Pena PA, Weier S *et al*. Stearidonic-enriched soybean oil modulates obesity, glucose metabolism, and fatty acid profiles independently of *Akkermansia muciniphila*. Mol Nutr Food Res. 2020;64:e2000162.
8. Pudlo NA, Urs K, Crawford R, Pirani A, Atherly T, Jimenez R *et al*. Phenotypic and genomic diversification in complex carbohydrate degrading human gut bacteria. bioRxiv. 2021;07.15.452266. doi: https://doi.org/10.1101/2021.07.15.452266

Supporting Figures and Tables

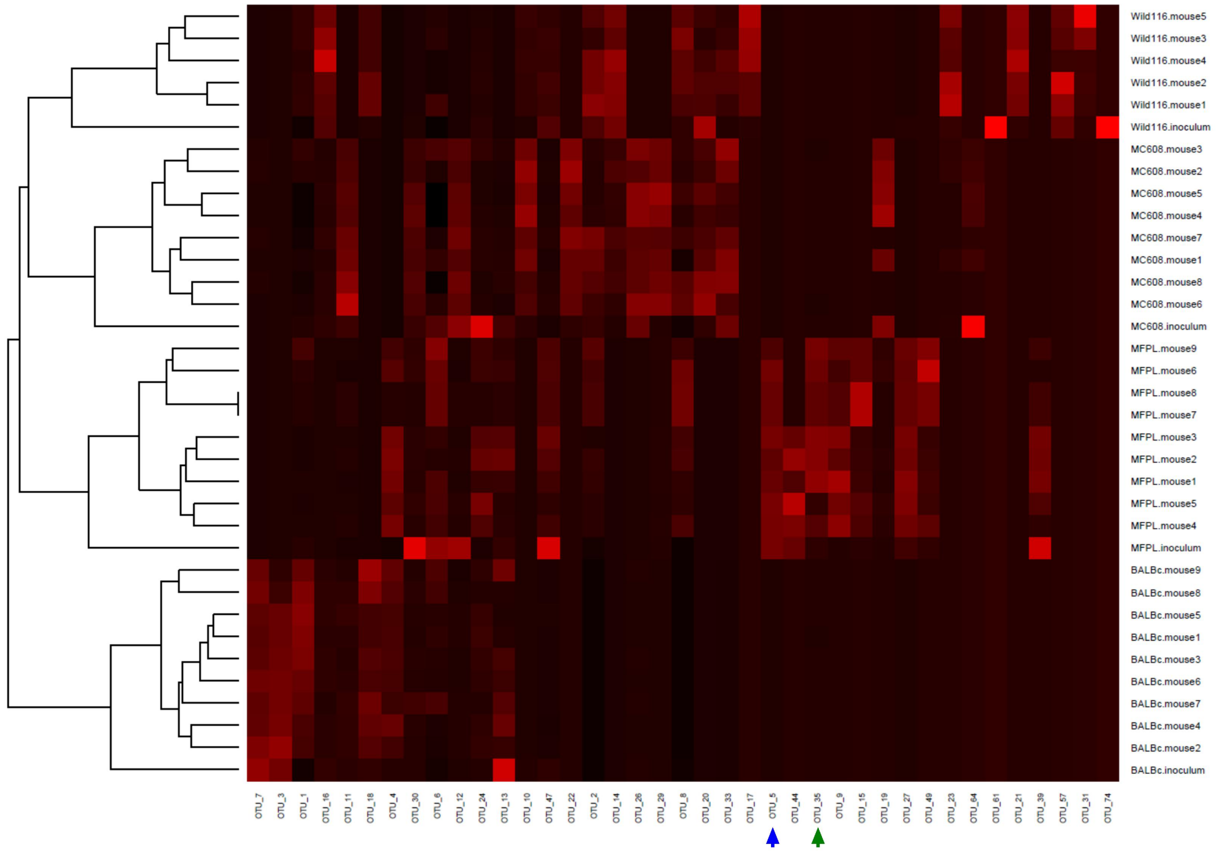

Fig S1. Complex microbiomes inoculated into germ-free mice established profiles similar to the donor inocula and are stable over four weeks. Germ-free mice were orally gavaged with one of four complex microbiomes (MFPL [4], MC608-F-a1 [4, 5], Wild116 [4-6], and BALBc.m3 [4, 7]), and fecal samples were collected from individual recipient mice four weeks after inoculation. Data are presented as the relative abundance (≥3%) obtained from 16S rRNA gene sequencing analysis. Hierarchical clustering was based on Bray-Curtis dissimilarities and hclust (average method). Only MFPL mice harbored both *A. muciniphila* and *B. vulgatus*. The MC608 microbiome was selected as the negative microbiome for our studies because it was naturally devoid of *A. muciniphila* and *B. vulgatus*. The blue arrow points to OTU_5, which yielded the closest blast hit to *A. muciniphila*. The green arrow points to OTU_35, which yielded the closest blast hit to *B. vulgatus*.

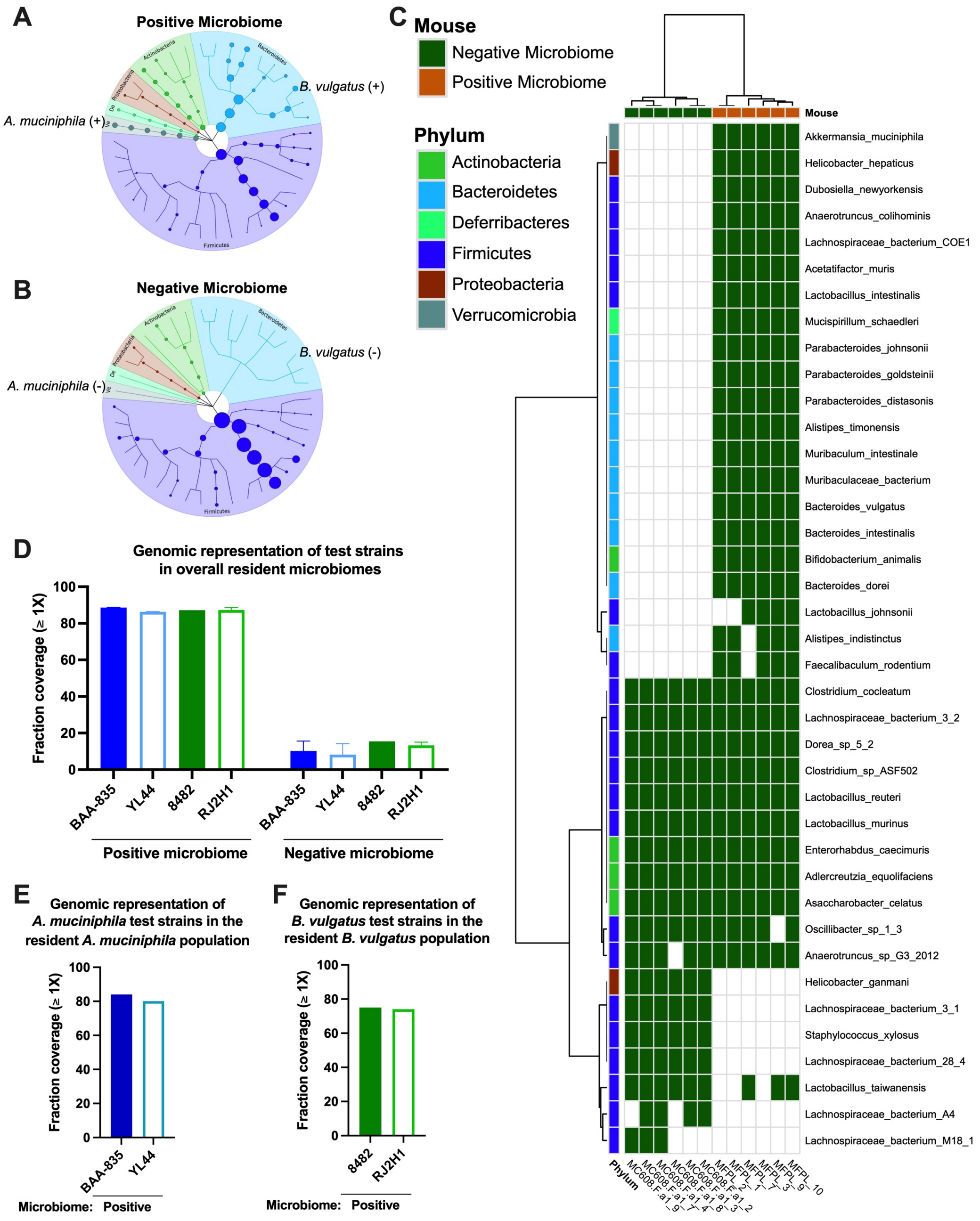

Fig S2. Composition and genomic representation of test strains in complex microbiomes. Compositional profiles for microbiomes with (positive; A) and without (negative; B) *A. muciniphila* and *B. vulgatus* were summarized and shown from phylum to the species levels. The abundance of reads mapping to a particular taxon is represented by the sizes of the circles on the phylogenetic trees. Ve = Verrucomicrobia; De = Deferribacteres. (C) Heatmap depicting presence/absence patterns of species (present in ≥ three mice in at least one treatment) in the positive (MFPL) and negative (MC608-F-a1) microbiomes as detected by MetaPhlAn3. (D) Fraction of the genome sequences from test strains that is represented in microbiomes. Fraction of the genome sequences from test strains that is represented in specific *B. vulgatus*-associated (E) and *A. muciniphila*-associated (F) contigs within the assembly of the positive microbiome.

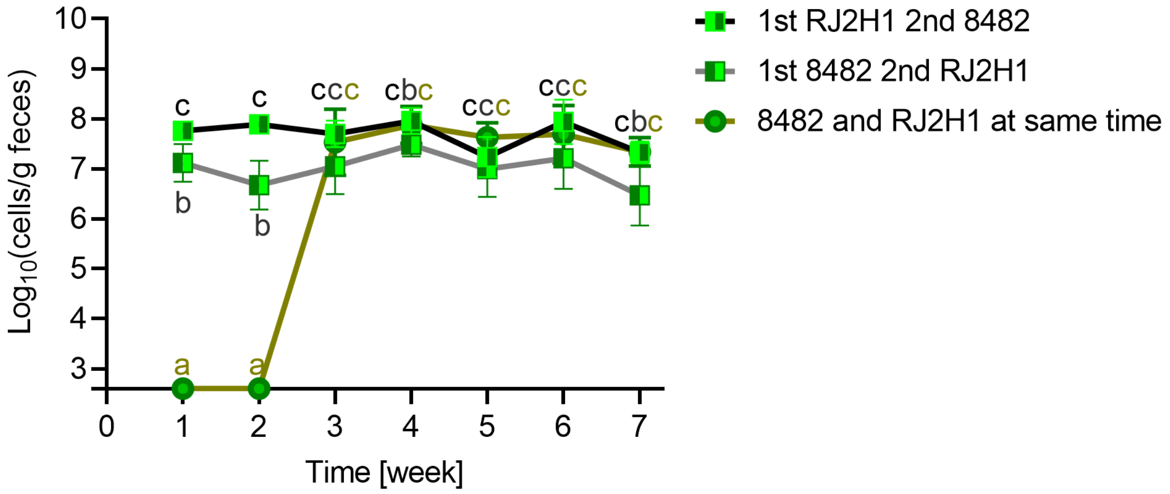

Fig S3. Total abundance of *B. vulgatus* (sum of the two strains) in mice colonized with *B. vulgatus* strains 8482 and RJ2H1 at various timepoints. Data are the sum of cell numbers of strains RJ2H1 and 8482 in fecal samples as determined by strain-specific qPCR from mice colonized first with RJ2H1 and then 8482 (black line), mice colonized first with 8482 and then RJ2H1 (gray line), and mice colonized with both *B. vulgatus* strains at the same time (gold line). Time points with the same letters are not significantly different from one another. Color of letters corresponds to the color of line representing each colonization scenario.

Table S1. Growth of *B. vulgatus* strains in medium with different carbohydrates. Data are expressed as the R^2 between replicate values and are published in Pudlo, Urs et al., 2021 [8].

Carbohydrate Substrates	Growth		Rate	
	8482	RJ2H1	8482	RJ2H1
Arabinan (sugar beet)	0.66	0.65	0.19	0.61
Arabinose	0.87	0.80	0.80	0.64
Fructose	1.00	0.77	0.90	0.41
Fucose	0.54	0.54	0.45	0.42
Galactose	0.58	0.91	0.50	0.97
Galacturonic acid	0.21	0.48	0.27	0.16
Glucose	0.98	0.67	0.88	0.33
Homogalacturonan (citrus peel)	0.30	0.24	0.18	0.18
Inulin (chicory root)	0.23	0.79	0.11	0.57
Mannose	0.85	0.62	0.79	0.59
mucin O-linked glycans (porcine gastric mucosa)	0.26	0.41	0.09	0.15
N-acetyl galactosamine	0.52	0.40	0.18	0.27
N-acetyl glucosamine	0.79	0.51	0.89	0.44
N-acetyl neuraminic acid	0.26	0.41	0.13	0.16
Pectic galactan (potato)	0.12	0.15	0.1	0.1
Pullulan (*Aureobasidium pullulans*)	0.09	1.00	0.23	0.8
Rhamnose	0.63	0.51	1.00	0.5
Xylose	0.93	0.98	1.00	1.00
Amylopectin (maize)	0	0.47	0	0.32
Amylopectin (potato)	0	0.32	0	0.3
Arabinogalactan (larch wood)	0	0.25	0	0.453
Chondroitin sulfate A/C (mixed isomers)	0.15	0	0.05	0
Glucosamine	0.17	0	0.06	0
Glycogen	0	0.78	0	0.8
Rhamnogalacturonan I (potato)	0	0.54	0	0.58
Xylan (water soluble from oat spelt)	0	0.11	0	0.12
Alginate	0	0	0	0
Arabinoxylan (wheat)	0	0	0	0
b-glucan (barley)	0	0	0	0
Cellobiose	0	0	0	0
Dextran (*Leuconostoc mesenteroides*)	0	0	0	0
Galactomannan (carob)	0	0	0	0
Glucomannan (konjac)	0	0	0	0
Glucuronic acid	0	0	0	0
Heparin (porcine mucosa)	0	0	0	0
Hyaluronan (cock's comb)	0	0	0	0
k-carrageenan	0	0	0	0
Laminarin	0	0	0	0
Levan (*Erwinia herbicola*)	0	0	0	0
Lichenin (Icelandic moss)	0	0	0	0
Pectic galactan (lupin)	0	0	0	0
Porphyran (uncooked, food grade *Porphyra* species)	0	0	0	0
Ribose	0	0	0	0
S. cerevisiae a-mannan	0	0	0	0
Xyloglucan (tamarind seed)	0	0	0	0

Table S2. *In vitro* test of antibiotics. TNTC refers to Too Numerous To Count.

**Antibiotic**	***B. vulgatus* 8482**	***B. vulgatus* RJ2H1**	***A. muciniphila* BAA-835**	***A. muciniphila* YL44**
Mix in media (# of colonies in plate)				
BHIym + 10 ug/mL Macrolide tylosin tartrate (MTT)	zero	zero	TNTC	TNTC
BHIym + 10 ug/mL Clarithromycin (Cla)	zero	zero	zero	zero
BHIym + 10 ug/mL Ampicillin sodium salt (AMP)	zero	zero	zero	zero
BHIym + 10 ug/mL each MTT+CLA+AMP	zero	zero	zero	zero
BHIym + 30 ug/mL Macrolide tylosin tartrate (MTT)	TNTC	TNTC	zero	zero
BHIym + 30 ug/mL Clarithromycin (CLA)	zero	20	zero	zero
BHIym + 30 ug/mL Ampicillin sodium salt (AMP)	zero	zero	zero	zero
BHIym + 30 ug/mL each MTT+CLA+AMP	zero	zero	zero	zero
Media w/o abx (positive control)	TNTC	TNTC	TNTC	TNTC
In Disc (mm radius of halo)				
Ampicillin	8	5	15	15
Kanamycin	No Halo	No Halo	No Halo	No Halo
Chloramphenicol	17	15	No Halo	No Halo
Streptomycin	No Halo	No Halo	No Halo	No Halo
Oxytetracycline	28	23	No Halo	No Halo
Erythromycin	18	18	No Halo	No Halo
